# 1′-(2-Chloro­phen­yl)-5,6,5′,6′,7′,7a′-hexa­hydro-1′*H*,1′′*H*-dispiro­[imidazo[2,1-*b*][1,3]thia­zole-2,2′-pyrrolizine-3′(2′*H*),3′′-indole]-2′′,3(2*H*,3′′*H*)-dione

**DOI:** 10.1107/S1600536810009323

**Published:** 2010-03-17

**Authors:** Bo Liao, Aiting Zheng, Bin Liu

**Affiliations:** a School of of Chemistry and Chemical Engineering, Hunan University of Science and Technology, Xiangtan 411201, People’s Republic of China

## Abstract

In the title compound, C_24_H_21_ClN_4_O_2_S, the two adjacent spiro junctions link an almost planar (r.m.s. deviation = 0.017 Å) 2-oxindole ring, a hexa­hydro-1*H*-pyrrolizine ring and a tetra­hydro­imidazo[2,1-*b*]thia­zole ring. In the crystal, inversion dimers linked by pairs of N—H⋯N hydrogen bonds occur, generating an *R*_2_^2^(16) loop.

## Related literature

For backgound to the properties of spiro-compounds, see: James *et al.* (1991 ▶); Kobayashi *et al.* (1991 ▶). For further synthetic details, see: Caramella & Grunanger (1984 ▶).
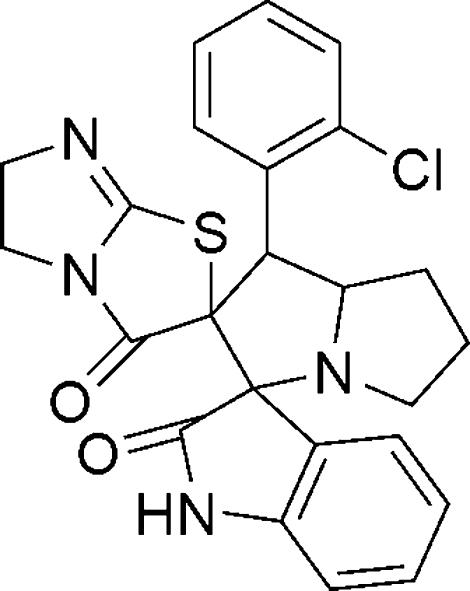


## Experimental

### 

#### Crystal data

C_24_H_21_ClN_4_O_2_S
*M* *_r_* = 464.96
Triclinic, 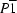

*a* = 8.6798 (18) Å
*b* = 11.078 (2) Å
*c* = 11.372 (2) Å
α = 78.984 (7)°
β = 81.867 (10)°
γ = 81.718 (11)°
*V* = 1054.8 (4) Å^3^
*Z* = 2
Cu *K*α radiation
μ = 2.78 mm^−1^
*T* = 113 K
0.28 × 0.24 × 0.20 mm

#### Data collection

Rigaku Saturn CCD area-detector diffractometer
Absorption correction: multi-scan (*CrystalClear*; Rigaku, 2001 ▶) *T*_min_ = 0.510, *T*_max_ = 0.606
21105 measured reflections
3986 independent reflections
3512 reflections with *I* > 2σ(*I*)
*R*_int_ = 0.075

#### Refinement

*R*[*F* ^2^ > 2σ(*F* ^2^)] = 0.043
*wR*(*F* ^2^) = 0.113
*S* = 1.02
3986 reflections
294 parameters
H atoms treated by a mixture of independent and constrained refinement
Δρ_max_ = 0.41 e Å^−3^
Δρ_min_ = −0.53 e Å^−3^

### 

Data collection: *CrystalClear* (Rigaku, 2001 ▶); cell refinement: *CrystalClear*; data reduction: *CrystalClear*; program(s) used to solve structure: *SHELXS97* (Sheldrick, 2008 ▶); program(s) used to refine structure: *SHELXL97* (Sheldrick, 2008 ▶); molecular graphics: *SHELXTL* (Sheldrick, 2008 ▶); software used to prepare material for publication: *SHELXL97*.

## Supplementary Material

Crystal structure: contains datablocks I, global. DOI: 10.1107/S1600536810009323/hb5356sup1.cif

Structure factors: contains datablocks I. DOI: 10.1107/S1600536810009323/hb5356Isup2.hkl

Additional supplementary materials:  crystallographic information; 3D view; checkCIF report

## Figures and Tables

**Table 1:** Hydrogen-bond geometry (Å, °)

*D*—H⋯*A*	*D*—H	H⋯*A*	*D*⋯*A*	*D*—H⋯*A*
N3—H1⋯N2^i^	0.88 (2)	2.10 (2)	2.971 (2)	170 (2)

Symmetry code: (i) 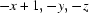.

**Fig. 1.**
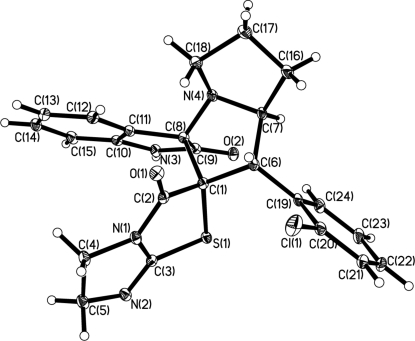
The molecular structure of (I), drawn with 30% probability ellipsoids.

**Fig. 2.**
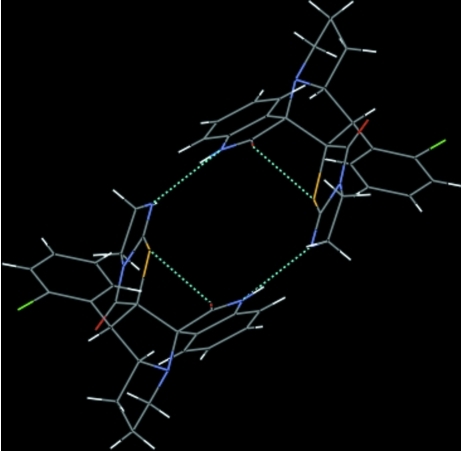
The hydrogen bonds of structure(I).

## supplementary crystallographic information

### Comment

Spiro-compounds represent an important class of naturally occurring substances, which in many cases exhibit important biological properties (Kobayashi *et al.*, 1991; James *et al.*, 1991). 1,3-Dipolar cycloaddition reactions are widely used for the construction of spiro-compounds (Caramella,1984). In this paper, the structure of the title compound is reported. The molecular structure of (I) is shown in Fig. 1. There exists a di-spiro ring system in the molecule which was consist of a 2-oxindole ring, a hexahydro-1*H*-pyrrolizine ring and a tetrahydroimidazo[2,1-b]thiazole ring. 2-oxindole ring (C8/C11/C12/C13/C14/C15/C10/N3/C9) is nearly planar that the mean deviation from this plane is 0.017Å. Two molecules are connected into a dimer by two N—H···N hydrogen bonds (Fig. 2).

### Experimental

A solution of 2-(2-chlorobenzylidene)-5,6-dihydroimidazo[2,1-b]thiazol-3(2*H*)-one (1 mmol), isatin (1 mmol) and proline (1 mmol) in methanol (30 ml) was refluxed overnight. Completion of the reaction was evidenced by TLC analysis. The solvent was removed in vacuo. The crude product was subjected to column chromatography using petroleum ether-ethyl acetate (v/v 5:1) as eluent to afford the title compound (I). m.p.463 K; ^1Ĥ-NMR (δ, p.p.m.): 1.80-1.84 (m, 3H), 2.13-2.20 (m, 2H), 2.77-2.80 (m, 1H), 3.44-3.47 (m, 1H), 3.55-3.57 (m, 1H), 3.90-3.92 (m, 2H), 3.98-4.00 (m, 1H), 4.64-4.66 (m, 1H), 6.83 (d, J = 7.5 Hz, 1H), 7.03-7.04 (m, 1H), 7.23-7.29 (m, 2H), 7.36-7.39 (m, 2H), 7.61-7.63 (m, 1H), 7.87-7.89 (m, 1H), 8.53 (bs, 1H); 20 mg of (I) was dissolved in 15 ml dioxane-ethyl acetate mixed solvent ; the solution was kept at room temperature for 15 d by natural evaporation to give colorless blocks of (I).

### Crystal data

**Table:**

C_24_H_21_ClN_4_O_2_S	*Z* = 2
*M**_r_* = 464.96	*F*(000) = 484
Triclinic, *P*1	*D*_x_ = 1.464 Mg m^−^^3^
Hall symbol: -P 1	Cu *K*α radiation, λ = 1.54187 Å
*a* = 8.6798 (18) Å	Cell parameters from 2350 reflections
*b* = 11.078 (2) Å	θ = 21.5–67.5°
*c* = 11.372 (2) Å	µ = 2.78 mm^−^^1^
α = 78.984 (7)°	*T* = 113 K
β = 81.867 (10)°	Block, colorless
γ = 81.718 (11)°	0.28 × 0.24 × 0.20 mm
*V* = 1054.8 (4) Å^3^	

### Data collection

**Table:**

Rigaku Saturn CCD area-detector diffractometer	3986 independent reflections
Radiation source: fine-focus sealed tube	3512 reflections with *I* > 2σ(*I*)
multilayer	*R*_int_ = 0.075
Detector resolution: 7.31 pixels mm^-1^	θ_max_ = 72.1°, θ_min_ = 4.0°
ω and φ scans	*h* = −9→10
Absorption correction: multi-scan (*CrystalClear*; Rigaku, 2001)	*k* = −13→13
*T*_min_ = 0.510, *T*_max_ = 0.606	*l* = −14→14
21105 measured reflections	

### Refinement

**Table:**

Refinement on *F*^2^	Secondary atom site location: difference Fourier map
Least-squares matrix: full	Hydrogen site location: inferred from neighbouring sites
*R*[*F*^2^ > 2σ(*F*^2^)] = 0.043	H atoms treated by a mixture of independent and constrained refinement
*wR*(*F*^2^) = 0.113	*w* = 1/[σ^2^(*F*_o_^2^) + (0.0802*P*)^2^] where *P* = (*F*_o_^2^ + 2*F*_c_^2^)/3
*S* = 1.02	(Δ/σ)_max_ < 0.001
3986 reflections	Δρ_max_ = 0.41 e Å^−^^3^
294 parameters	Δρ_min_ = −0.53 e Å^−^^3^
0 restraints	Extinction correction: *SHELXL97* (Sheldrick, 2008), Fc^*^=kFc[1+0.001xFc^2^λ^3^/sin(2θ)]^-1/4^
Primary atom site location: structure-invariant direct methods	Extinction coefficient: 0.0128 (10)

### Special details

**Table:**

Geometry. All esds (except the esd in the dihedral angle between two l.s. planes) are estimated using the full covariance matrix. The cell esds are taken into account individually in the estimation of esds in distances, angles and torsion angles; correlations between esds in cell parameters are only used when they are defined by crystal symmetry. An approximate (isotropic) treatment of cell esds is used for estimating esds involving l.s. planes.
Refinement. Refinement of *F*^2^ against ALL reflections. The weighted *R*-factor wR and goodness of fit *S* are based on *F*^2^, conventional *R*-factors *R* are based on *F*, with *F* set to zero for negative *F*^2^. The threshold expression of *F*^2^ > σ(*F*^2^) is used only for calculating *R*-factors(gt) etc. and is not relevant to the choice of reflections for refinement. *R*-factors based on *F*^2^ are statistically about twice as large as those based on *F*, and *R*- factors based on ALL data will be even larger.

### Fractional atomic coordinates and isotropic or equivalent isotropic displacement parameters (Å^2^)

**Table:**

	*x*	*y*	*z*	*U*_iso_*/*U*_eq_	
Cl1	0.24861 (7)	0.40648 (4)	0.48838 (4)	0.03409 (17)	
S1	0.43258 (5)	0.19148 (3)	0.16037 (3)	0.01711 (15)	
O1	0.13769 (14)	0.10571 (11)	0.44297 (10)	0.0206 (3)	
O2	0.21446 (14)	0.28209 (11)	−0.03632 (10)	0.0211 (3)	
N1	0.37173 (17)	0.02732 (13)	0.35068 (12)	0.0179 (3)	
N2	0.60368 (17)	−0.02950 (13)	0.24368 (12)	0.0202 (3)	
N3	0.20977 (17)	0.07131 (13)	−0.01187 (12)	0.0182 (3)	
N4	−0.03261 (16)	0.24825 (12)	0.17028 (12)	0.0174 (3)	
C1	0.22952 (19)	0.20199 (14)	0.23784 (13)	0.0150 (3)	
C2	0.2381 (2)	0.10826 (15)	0.35725 (13)	0.0162 (3)	
C3	0.4828 (2)	0.05014 (15)	0.25168 (13)	0.0165 (3)	
C4	0.4173 (2)	−0.09606 (15)	0.41941 (15)	0.0219 (4)	
H4A	0.3472	−0.1565	0.4119	0.026*	
H4B	0.4192	−0.0936	0.5058	0.026*	
C5	0.5847 (2)	−0.12500 (16)	0.35418 (15)	0.0228 (4)	
H5A	0.6644	−0.1226	0.4076	0.027*	
H5B	0.5979	−0.2086	0.3324	0.027*	
C6	0.15375 (19)	0.33307 (14)	0.26047 (14)	0.0163 (3)	
H6	0.1057	0.3231	0.3465	0.020*	
C7	0.0155 (2)	0.36758 (15)	0.18111 (14)	0.0179 (3)	
H7	0.0509	0.4145	0.0999	0.021*	
C8	0.11006 (19)	0.16210 (15)	0.15955 (13)	0.0150 (3)	
C9	0.18421 (19)	0.18303 (15)	0.02409 (14)	0.0169 (3)	
C10	0.15500 (19)	−0.02286 (15)	0.07815 (14)	0.0165 (3)	
C11	0.09088 (19)	0.02528 (15)	0.18252 (14)	0.0160 (3)	
C12	0.0259 (2)	−0.05294 (15)	0.28191 (14)	0.0190 (4)	
H12	−0.0177	−0.0220	0.3533	0.023*	
C13	0.0256 (2)	−0.17737 (16)	0.27560 (16)	0.0217 (4)	
H13	−0.0187	−0.2314	0.3431	0.026*	
C14	0.0894 (2)	−0.22283 (16)	0.17121 (16)	0.0228 (4)	
H14	0.0880	−0.3078	0.1684	0.027*	
C15	0.1554 (2)	−0.14631 (15)	0.07089 (15)	0.0214 (4)	
H15	0.1992	−0.1777	−0.0003	0.026*	
C16	−0.1309 (2)	0.43859 (16)	0.24054 (16)	0.0237 (4)	
H16A	−0.1688	0.5132	0.1843	0.028*	
H16B	−0.1082	0.4641	0.3145	0.028*	
C17	−0.2531 (2)	0.34589 (17)	0.27121 (18)	0.0287 (4)	
H17A	−0.3134	0.3511	0.3511	0.034*	
H17B	−0.3268	0.3617	0.2095	0.034*	
C18	−0.1555 (2)	0.21991 (16)	0.27193 (16)	0.0220 (4)	
H18A	−0.1101	0.1902	0.3489	0.026*	
H18B	−0.2182	0.1573	0.2577	0.026*	
C19	0.2614 (2)	0.43184 (15)	0.24310 (14)	0.0179 (3)	
C20	0.3102 (2)	0.47270 (15)	0.33957 (15)	0.0208 (4)	
C21	0.4077 (2)	0.56475 (16)	0.32325 (17)	0.0261 (4)	
H21	0.4377	0.5904	0.3909	0.031*	
C22	0.4612 (2)	0.61903 (16)	0.20712 (18)	0.0266 (4)	
H22	0.5288	0.6817	0.1945	0.032*	
C23	0.4147 (2)	0.58077 (16)	0.10959 (16)	0.0253 (4)	
H23	0.4500	0.6179	0.0298	0.030*	
C24	0.3173 (2)	0.48903 (16)	0.12801 (15)	0.0222 (4)	
H24	0.2874	0.4641	0.0599	0.027*	
H1	0.255 (3)	0.064 (2)	−0.085 (2)	0.032 (6)*	

### Atomic displacement parameters (Å^2^)

**Table:**

	*U*^11^	*U*^22^	*U*^33^	*U*^12^	*U*^13^	*U*^23^
Cl1	0.0545 (4)	0.0322 (3)	0.0213 (2)	−0.0151 (2)	−0.0128 (2)	−0.00515 (18)
S1	0.0177 (2)	0.0147 (2)	0.0184 (2)	0.00019 (16)	−0.00128 (15)	−0.00395 (15)
O1	0.0237 (7)	0.0229 (6)	0.0163 (6)	−0.0034 (5)	−0.0004 (5)	−0.0073 (4)
O2	0.0280 (7)	0.0175 (6)	0.0183 (6)	−0.0026 (5)	−0.0036 (5)	−0.0037 (4)
N1	0.0221 (8)	0.0151 (7)	0.0167 (6)	0.0001 (6)	−0.0035 (5)	−0.0039 (5)
N2	0.0233 (8)	0.0173 (7)	0.0213 (7)	0.0020 (6)	−0.0052 (6)	−0.0083 (5)
N3	0.0240 (8)	0.0164 (7)	0.0156 (7)	0.0008 (6)	−0.0024 (5)	−0.0089 (5)
N4	0.0185 (7)	0.0142 (7)	0.0213 (7)	0.0019 (6)	−0.0037 (5)	−0.0096 (5)
C1	0.0170 (8)	0.0151 (8)	0.0142 (7)	−0.0015 (6)	−0.0004 (6)	−0.0073 (6)
C2	0.0214 (9)	0.0145 (8)	0.0156 (7)	−0.0035 (6)	−0.0042 (6)	−0.0070 (6)
C3	0.0202 (9)	0.0148 (8)	0.0172 (7)	−0.0017 (7)	−0.0049 (6)	−0.0079 (6)
C4	0.0292 (10)	0.0141 (8)	0.0223 (8)	0.0004 (7)	−0.0072 (7)	−0.0024 (6)
C5	0.0290 (10)	0.0166 (8)	0.0237 (8)	0.0030 (7)	−0.0080 (7)	−0.0065 (6)
C6	0.0190 (9)	0.0141 (8)	0.0176 (7)	−0.0007 (6)	−0.0021 (6)	−0.0082 (6)
C7	0.0209 (9)	0.0124 (8)	0.0219 (8)	0.0007 (7)	−0.0045 (6)	−0.0076 (6)
C8	0.0171 (8)	0.0154 (8)	0.0144 (7)	−0.0006 (6)	−0.0024 (6)	−0.0078 (5)
C9	0.0184 (8)	0.0176 (8)	0.0162 (7)	0.0018 (6)	−0.0055 (6)	−0.0075 (6)
C10	0.0159 (8)	0.0174 (8)	0.0181 (7)	0.0017 (6)	−0.0050 (6)	−0.0081 (6)
C11	0.0166 (8)	0.0148 (8)	0.0190 (7)	0.0010 (6)	−0.0050 (6)	−0.0089 (6)
C12	0.0204 (9)	0.0185 (8)	0.0200 (8)	−0.0012 (7)	−0.0036 (6)	−0.0080 (6)
C13	0.0205 (9)	0.0179 (8)	0.0270 (8)	−0.0030 (7)	−0.0037 (7)	−0.0033 (6)
C14	0.0240 (10)	0.0138 (8)	0.0335 (9)	0.0013 (7)	−0.0082 (7)	−0.0101 (7)
C15	0.0253 (9)	0.0178 (8)	0.0238 (8)	0.0037 (7)	−0.0063 (7)	−0.0126 (6)
C16	0.0228 (9)	0.0187 (9)	0.0319 (9)	0.0035 (7)	−0.0052 (7)	−0.0130 (7)
C17	0.0216 (10)	0.0236 (10)	0.0416 (10)	0.0026 (8)	−0.0005 (8)	−0.0139 (8)
C18	0.0189 (9)	0.0194 (8)	0.0289 (9)	−0.0003 (7)	−0.0008 (7)	−0.0101 (6)
C19	0.0200 (9)	0.0116 (7)	0.0238 (8)	0.0028 (6)	−0.0037 (6)	−0.0101 (6)
C20	0.0238 (9)	0.0159 (8)	0.0251 (8)	0.0012 (7)	−0.0077 (7)	−0.0082 (6)
C21	0.0259 (10)	0.0185 (9)	0.0381 (10)	0.0006 (7)	−0.0108 (8)	−0.0127 (7)
C22	0.0226 (10)	0.0163 (9)	0.0438 (11)	−0.0034 (7)	−0.0020 (8)	−0.0132 (7)
C23	0.0277 (10)	0.0172 (8)	0.0302 (9)	−0.0021 (7)	0.0045 (7)	−0.0083 (7)
C24	0.0271 (10)	0.0167 (8)	0.0242 (8)	0.0009 (7)	−0.0016 (7)	−0.0106 (6)

### Geometric parameters (Å, °)

**Table:**

Cl1—C20	1.7522 (18)	C8—C9	1.571 (2)
S1—C3	1.7407 (16)	C10—C15	1.385 (2)
S1—C1	1.8539 (17)	C10—C11	1.404 (2)
O1—C2	1.211 (2)	C11—C12	1.390 (2)
O2—C9	1.220 (2)	C12—C13	1.394 (2)
N1—C2	1.363 (2)	C12—H12	0.9500
N1—C3	1.384 (2)	C13—C14	1.390 (2)
N1—C4	1.469 (2)	C13—H13	0.9500
N2—C3	1.275 (2)	C14—C15	1.390 (3)
N2—C5	1.486 (2)	C14—H14	0.9500
N3—C9	1.355 (2)	C15—H15	0.9500
N3—C10	1.400 (2)	C16—C17	1.540 (3)
N3—H1	0.88 (2)	C16—H16A	0.9900
N4—C8	1.456 (2)	C16—H16B	0.9900
N4—C7	1.474 (2)	C17—C18	1.524 (2)
N4—C18	1.477 (2)	C17—H17A	0.9900
C1—C2	1.548 (2)	C17—H17B	0.9900
C1—C6	1.558 (2)	C18—H18A	0.9900
C1—C8	1.614 (2)	C18—H18B	0.9900
C4—C5	1.555 (3)	C19—C24	1.394 (2)
C4—H4A	0.9900	C19—C20	1.401 (2)
C4—H4B	0.9900	C20—C21	1.387 (3)
C5—H5A	0.9900	C21—C22	1.387 (3)
C5—H5B	0.9900	C21—H21	0.9500
C6—C19	1.507 (2)	C22—C23	1.389 (3)
C6—C7	1.563 (2)	C22—H22	0.9500
C6—H6	1.0000	C23—C24	1.381 (3)
C7—C16	1.537 (2)	C23—H23	0.9500
C7—H7	1.0000	C24—H24	0.9500
C8—C11	1.517 (2)		
			
C3—S1—C1	91.34 (8)	N3—C9—C8	107.47 (13)
C2—N1—C3	118.08 (13)	C15—C10—N3	127.81 (15)
C2—N1—C4	132.63 (14)	C15—C10—C11	121.99 (16)
C3—N1—C4	108.49 (13)	N3—C10—C11	110.17 (14)
C3—N2—C5	105.37 (14)	C12—C11—C10	119.27 (15)
C9—N3—C10	112.22 (13)	C12—C11—C8	132.62 (14)
C9—N3—H1	120.9 (15)	C10—C11—C8	108.11 (14)
C10—N3—H1	126.9 (15)	C11—C12—C13	119.24 (15)
C8—N4—C7	106.79 (13)	C11—C12—H12	120.4
C8—N4—C18	119.35 (13)	C13—C12—H12	120.4
C7—N4—C18	106.15 (12)	C14—C13—C12	120.45 (17)
C2—C1—C6	111.70 (12)	C14—C13—H13	119.8
C2—C1—C8	109.24 (13)	C12—C13—H13	119.8
C6—C1—C8	103.51 (12)	C13—C14—C15	121.26 (16)
C2—C1—S1	104.85 (11)	C13—C14—H14	119.4
C6—C1—S1	116.29 (11)	C15—C14—H14	119.4
C8—C1—S1	111.25 (10)	C10—C15—C14	117.79 (15)
O1—C2—N1	125.06 (15)	C10—C15—H15	121.1
O1—C2—C1	124.45 (15)	C14—C15—H15	121.1
N1—C2—C1	110.46 (13)	C7—C16—C17	104.65 (13)
N2—C3—N1	117.43 (14)	C7—C16—H16A	110.8
N2—C3—S1	130.89 (13)	C17—C16—H16A	110.8
N1—C3—S1	111.62 (12)	C7—C16—H16B	110.8
N1—C4—C5	99.92 (13)	C17—C16—H16B	110.8
N1—C4—H4A	111.8	H16A—C16—H16B	108.9
C5—C4—H4A	111.8	C18—C17—C16	104.10 (15)
N1—C4—H4B	111.8	C18—C17—H17A	110.9
C5—C4—H4B	111.8	C16—C17—H17A	110.9
H4A—C4—H4B	109.5	C18—C17—H17B	110.9
N2—C5—C4	107.58 (13)	C16—C17—H17B	110.9
N2—C5—H5A	110.2	H17A—C17—H17B	109.0
C4—C5—H5A	110.2	N4—C18—C17	101.57 (14)
N2—C5—H5B	110.2	N4—C18—H18A	111.5
C4—C5—H5B	110.2	C17—C18—H18A	111.5
H5A—C5—H5B	108.5	N4—C18—H18B	111.5
C19—C6—C1	117.03 (14)	C17—C18—H18B	111.5
C19—C6—C7	113.84 (13)	H18A—C18—H18B	109.3
C1—C6—C7	105.08 (12)	C24—C19—C20	116.04 (16)
C19—C6—H6	106.8	C24—C19—C6	121.04 (15)
C1—C6—H6	106.8	C20—C19—C6	122.91 (15)
C7—C6—H6	106.8	C21—C20—C19	122.76 (17)
N4—C7—C16	105.48 (14)	C21—C20—Cl1	117.18 (13)
N4—C7—C6	105.24 (12)	C19—C20—Cl1	120.06 (14)
C16—C7—C6	113.85 (13)	C20—C21—C22	119.36 (17)
N4—C7—H7	110.7	C20—C21—H21	120.3
C16—C7—H7	110.7	C22—C21—H21	120.3
C6—C7—H7	110.7	C21—C22—C23	119.26 (18)
N4—C8—C11	116.83 (14)	C21—C22—H22	120.4
N4—C8—C9	108.11 (12)	C23—C22—H22	120.4
C11—C8—C9	101.84 (12)	C24—C23—C22	120.37 (17)
N4—C8—C1	106.18 (12)	C24—C23—H23	119.8
C11—C8—C1	115.78 (12)	C22—C23—H23	119.8
C9—C8—C1	107.48 (13)	C23—C24—C19	122.21 (16)
O2—C9—N3	126.81 (15)	C23—C24—H24	118.9
O2—C9—C8	125.72 (14)	C19—C24—H24	118.9
			
C3—S1—C1—C2	17.34 (11)	C2—C1—C8—C9	−139.50 (13)
C3—S1—C1—C6	141.23 (11)	C6—C1—C8—C9	101.38 (13)
C3—S1—C1—C8	−100.62 (11)	S1—C1—C8—C9	−24.22 (14)
C3—N1—C2—O1	−173.38 (15)	C10—N3—C9—O2	−177.34 (16)
C4—N1—C2—O1	18.2 (3)	C10—N3—C9—C8	3.48 (18)
C3—N1—C2—C1	8.4 (2)	N4—C8—C9—O2	52.9 (2)
C4—N1—C2—C1	−159.95 (16)	C11—C8—C9—O2	176.57 (16)
C6—C1—C2—O1	37.6 (2)	C1—C8—C9—O2	−61.3 (2)
C8—C1—C2—O1	−76.26 (19)	N4—C8—C9—N3	−127.87 (14)
S1—C1—C2—O1	164.42 (14)	C11—C8—C9—N3	−4.24 (16)
C6—C1—C2—N1	−144.15 (14)	C1—C8—C9—N3	117.90 (14)
C8—C1—C2—N1	101.94 (15)	C9—N3—C10—C15	176.92 (16)
S1—C1—C2—N1	−17.38 (15)	C9—N3—C10—C11	−1.15 (19)
C5—N2—C3—N1	−1.89 (19)	C15—C10—C11—C12	0.1 (2)
C5—N2—C3—S1	175.10 (13)	N3—C10—C11—C12	178.33 (14)
C2—N1—C3—N2	−176.64 (14)	C15—C10—C11—C8	179.98 (14)
C4—N1—C3—N2	−5.6 (2)	N3—C10—C11—C8	−1.82 (18)
C2—N1—C3—S1	5.81 (19)	N4—C8—C11—C12	−59.1 (2)
C4—N1—C3—S1	176.82 (11)	C9—C8—C11—C12	−176.61 (17)
C1—S1—C3—N2	168.82 (16)	C1—C8—C11—C12	67.1 (2)
C1—S1—C3—N1	−14.05 (12)	N4—C8—C11—C10	121.09 (14)
C2—N1—C4—C5	178.86 (16)	C9—C8—C11—C10	3.57 (16)
C3—N1—C4—C5	9.66 (16)	C1—C8—C11—C10	−112.67 (15)
C3—N2—C5—C4	8.22 (18)	C10—C11—C12—C13	−0.2 (2)
N1—C4—C5—N2	−10.75 (16)	C8—C11—C12—C13	−179.98 (16)
C2—C1—C6—C19	107.20 (16)	C11—C12—C13—C14	0.0 (3)
C8—C1—C6—C19	−135.39 (13)	C12—C13—C14—C15	0.1 (3)
S1—C1—C6—C19	−13.08 (17)	N3—C10—C15—C14	−177.82 (16)
C2—C1—C6—C7	−125.40 (14)	C11—C10—C15—C14	0.0 (2)
C8—C1—C6—C7	−7.99 (15)	C13—C14—C15—C10	−0.2 (3)
S1—C1—C6—C7	114.32 (12)	N4—C7—C16—C17	5.22 (17)
C8—N4—C7—C16	−158.43 (12)	C6—C7—C16—C17	−109.65 (16)
C18—N4—C7—C16	−30.09 (16)	C7—C16—C17—C18	20.32 (18)
C8—N4—C7—C6	−37.75 (15)	C8—N4—C18—C17	163.22 (14)
C18—N4—C7—C6	90.59 (14)	C7—N4—C18—C17	42.69 (16)
C19—C6—C7—N4	156.75 (13)	C16—C17—C18—N4	−38.17 (17)
C1—C6—C7—N4	27.44 (16)	C1—C6—C19—C24	76.18 (19)
C19—C6—C7—C16	−88.23 (17)	C7—C6—C19—C24	−46.8 (2)
C1—C6—C7—C16	142.45 (14)	C1—C6—C19—C20	−104.47 (18)
C7—N4—C8—C11	163.22 (13)	C7—C6—C19—C20	132.52 (16)
C18—N4—C8—C11	43.02 (19)	C24—C19—C20—C21	0.2 (2)
C7—N4—C8—C9	−82.74 (14)	C6—C19—C20—C21	−179.17 (15)
C18—N4—C8—C9	157.06 (14)	C24—C19—C20—Cl1	−179.45 (12)
C7—N4—C8—C1	32.35 (15)	C6—C19—C20—Cl1	1.2 (2)
C18—N4—C8—C1	−87.84 (16)	C19—C20—C21—C22	−0.4 (3)
C2—C1—C8—N4	104.98 (14)	Cl1—C20—C21—C22	179.24 (13)
C6—C1—C8—N4	−14.14 (15)	C20—C21—C22—C23	0.6 (3)
S1—C1—C8—N4	−139.74 (11)	C21—C22—C23—C24	−0.5 (3)
C2—C1—C8—C11	−26.48 (18)	C22—C23—C24—C19	0.3 (3)
C6—C1—C8—C11	−145.59 (13)	C20—C19—C24—C23	−0.1 (2)
S1—C1—C8—C11	88.80 (14)	C6—C19—C24—C23	179.24 (16)

### Hydrogen-bond geometry (Å, °)

**Table:**

*D*—H···*A*	*D*—H	H···*A*	*D*···*A*	*D*—H···*A*
N3—H1···N2^i^	0.88 (2)	2.10 (2)	2.971 (2)	170 (2)

Symmetry codes: (i) −*x*+1, −*y*, −*z*.
